# Controlling the stoichiometry and strand polarity of a tetramolecular G-quadruplex structure by using a DNA origami frame

**DOI:** 10.1093/nar/gkt592

**Published:** 2013-07-17

**Authors:** Arivazhagan Rajendran, Masayuki Endo, Kumi Hidaka, Phong Lan Thao Tran, Jean-Louis Mergny, Hiroshi Sugiyama

**Affiliations:** ^1^Department of Chemistry, Graduate School of Science, Kyoto University, Kitashirakawa-oiwakecho, Sakyo-ku, Kyoto 606-8502, Japan, ^2^Institute for Integrated Cell-Material Sciences (WPI-iCeMS), Kyoto University, Yoshida-ushinomiyacho, Sakyo-ku, Kyoto 606-8501, Japan, ^3^CREST, Japan Science and Technology Corporation (JST), Sanbancho, Chiyoda-ku, Tokyo 102-0075, Japan and ^4^University of Bordeaux, INSERM, U869, ARNA Laboratory, 2 rue Robert Escarpit, Pessac, F-33607, France

## Abstract

Guanine-rich oligonucleotides often show a strong tendency to form supramolecular architecture, the so-called G-quadruplex structure. Because of the biological significance, it is now considered to be one of the most important conformations of DNA. Here, we describe the direct visualization and single-molecule analysis of the formation of a tetramolecular G-quadruplex in KCl solution. The conformational changes were carried out by incorporating two duplex DNAs, with G–G mismatch repeats in the middle, inside a DNA origami frame and monitoring the topology change of the strands. In the absence of KCl, incorporated duplexes had no interaction and laid parallel to each other. Addition of KCl induced the formation of a G-quadruplex structure by stably binding the duplexes to each other in the middle. Such a quadruplex formation allowed the DNA synapsis without disturbing the duplex regions of the participating sequences, and resulted in an X-shaped structure that was monitored by atomic force microscopy. Further, the G-quadruplex formation in KCl solution and its disruption in KCl-free buffer were analyzed in real-time. The orientation of the G-quadruplex is often difficult to control and investigate using traditional biochemical methods. However, our method using DNA origami could successfully control the strand orientations, topology and stoichiometry of the G-quadruplex.

## INTRODUCTION

Nucleic acids can adopt structures other than the canonical B-form duplex stabilized by Watson–Crick base pairing (1–4). Among the noncanonical secondary structures (5–10), the G-quadruplex is an attractive conformation of nucleic acids, and structural studies of G-quadruplex motifs are ongoing tasks in the field of chemical biology of nucleic acids (5,10–12). G-quadruplex is a supramolecular architecture, which represents an unusual DNA secondary structure that can be formed by certain G-rich sequences. These structures comprise highly stable planar rings of four guanines stabilized through Hoogsteen hydrogen bonds leading to the formation of G-quartets (13). The stacking of the formed quartets contributes substantially to the stability of the quadruplex structure. The minimum requirement for the formation of a quadruplex is four separate tracts of at least three consecutive guanines. The topology of the G-quadruplex structures is polymorphic and reflects a range of alternate strand directionalities, loop connectivities and *syn*/*anti*-distribution of guanine bases around G-tetrads. This polymorphism and the stability of the quadruplex structures depend on the nucleic acid sequence, stacking between different G-tetrads, temperature, solvent, electrostatic interactions mediated by salts, and salt composition (14). For instance, it has been proposed that the quadruplex structure is regulated by sodium–potassium exchange (15). In addition to the strand polarity (parallel, antiparallel or mixed), glycosidic torsion angle (*syn* or *anti*) and the orientation of the loops (lateral, diagonal or both) (16), the G-quadruplex structures can also be differentiated by considering the stoichiometry of the strands into one (exclusively intramolecular structure) (5), two [for example, (3 + 1) type structure] (17), three (as we have described recently) (18) or four strands (19) (described in this report).

Although G-quadruplex structures were first observed with guanosine mononucleotides, and synthetic G-rich oligomers (13), they were later found in natural sequences such as human chromosomal telomeres comprising the tandem repeats of TTAGGG sequence with a single-stranded 3′-overhang of 100–200 nucleotides (20). Apart from the telomeric repeat sequences, the quadruplex-forming G-rich regions have been identified in the human genome with enrichment in promoter regions of several proto-oncogenes such as *c-myc*, *c-kit* and *K-ras* (12,21–23). There are experimental evidences for the G-quadruplex formation in the genomic DNA of several other organisms (24–26). The formation of the quadruplex can affect a wide range of biological activities including genome stability, cell growth, gene regulation, transcription, translation, DNA replication and DNA repair (27). Thus, G-rich regions are of great interest for therapeutic targets such as developing anticancer agents (28–31).

We have recently reported the real-time analyses on a (3 + 1) type G-quadruplex structure (17) and a theoretical analysis of the folding pathways of human telomeric type-1 and type-2 G-quadruplex structures (5). In this report, we present the direct and real-time analysis on the salt-induced formation of a tetramolecular G-quadruplex structure and its disruption in a salt-free condition. The materials prepared by the scaffolded DNA origami method (32–36) are shown to be novel substrates for the analysis of single-molecule reactions and functions (37–45). Thus, we have performed our analyses within a nanovessel (46,47) constructed using the origami method. Cations such as K^+^ and Na^+^ are known to stabilize the G-quadruplex structures via binding at the center of the G-quadruplex, generally sandwiched between two consecutive quartets (48). Because of the high intracellular concentration of K^+^, we have used K^+^ ions to mimic the cell-like condition and to execute the conformational switching. The conformational switching was monitored using high-speed atomic force microscopy (HS-AFM) (49–54) by following the topology change of the strands.

The important achievement in this study over the previous reports (17,19) is the demonstration of the capabilities of DNA origami structures for the control over the stoichiometry and strand polarity of the G-quadruplex structures. A number of indirect techniques have been used to study quadruplex structures. Among them, circular dichroism, thermal difference spectroscopy and gel electrophoresis have been used extensively. However, these techniques often fail to provide sufficient information about the strand polarity and stoichiometry. For example, Sen *et al.* reported a similar DNA synapsis through the formation of a tetramolecular G-quadruplex using gel electrophoresis (19). Though they could successfully achieve the DNA synapsis through the quadruplex formation, they found a mixture of quadruplex structures with different stoichiometry of the strands. However, in our single-molecule analysis using DNA origami, we could successfully control the stoichiometry of the strands and study exclusively the single G-quadruplex of interest rather than a mixture. Moreover, we have attached the DNA strands of interest within a DNA origami frame and we could successfully control the strand polarity (in the present case, antiparallel or mixed conformation), whereas it is difficult to control using other techniques or methods of analysis.

## MATERIALS AND METHODS

### Chemicals and reagents

Tris–HCl, EDTA, MgCl_2_ and KCl were purchased from Nacalai Tesque, Inc. (Kyoto, Japan). Single-stranded M13mp18 DNA was obtained from New England Biolabs, Inc. (Ipswich, MA, USA; catalog no. N4040S). The staple strands (most of them are 32-mer) for the fabrication of the DNA origami frame (46), and the oligomers for the synaptic DNA were received from Sigma Genosys (Hokkaido, Japan). The gel-filtration column and sephacryl S-300 were purchased from Bio-Rad Laboratories, Inc. (Hercules, CA, USA) and GE Healthcare UK Ltd. (Buckinghamshire, UK), respectively. Water was deionized (≥18.0 MΩ cm specific resistance at 25°C) by a Milli-Q system (Millipore Corp., Bedford, MA, USA).

### Preparation of the origami frame and incorporation of the duplexes

Origami frame was prepared by annealing the solution of M13mp18 DNA (final concentration of 0.01 µM), staple DNA strands (4 equivalent 0.04 µM), Tris–HCl (20 mM, pH 7.6), EDTA (1 mM), MgCl_2_ (10 mM) and KCl (0 or 100 mM) from 85 to 15°C at a rate of −1°C/min (33). The duplex DNAs (final concentration of 0.1 µM each) were also prepared using the same condition with that of the origami frame. Ten-fold excess of each duplex was then mixed with the origami frame. The self-assembly of these duplexes inside the origami frame was carried out by reannealing the solution from 50 to 15°C at a rate of −1°C/min. The duplexes-incorporated origami was purified using sephacryl S-300 gel-filtration column before HS-AFM imaging. Gel-filtration columns were prepared in the same amount of buffer and salt with that of the origami solution that has to be purified. After purification, yield of the origami formation and incorporation of the duplexes inside the origami frame was found to be ∼100%, while <10% of the structures were broken during the AFM scanning. For the analysis in the absence of K^+^, all the experimental steps such as preparation of origami and duplex strands, sephacryl column, surface immobilization and observation buffer contained no KCl, while all these steps contained 100 mM KCl for the experiments in the presence of K^+^.

### AFM imaging

AFM images were recorded using a fast-scanning AFM system (46,50,55–57) (Nano Live Vision, RIBM Co. Ltd., Tsukuba, Japan) with a silicon nitride cantilever (resonant frequency 1–2 MHz, spring constant 0.1–0.3 N/m, EBDTip radius <15 nm, Olympus BL-AC10EGS-A2). The sample (2 µl) was adsorbed onto a freshly cleaved mica plate (ϕ 1.5 mm, RIBM Co. Ltd., Tsukuba, Japan) for 5 min at room temperature and then the surface was washed three to five times using the same buffer solution with same concentration of KCl with that of the origami was prepared. Scanning was performed using the tapping mode in the same buffer solution. AFM images were recorded either using the observation buffer that contained KCl or KCl-free buffer depending on the requirements. All images reported here were recorded with a scan speed of 0.2 frame/s. The yield calculations of the parallel- and X-shapes were carried out by counting the shapes in the AFM images. The broken structures were not taken into account for the yield calculations.

## RESULTS AND DISCUSSION

### Design of the origami frame and duplex DNAs

**Figure 1.**
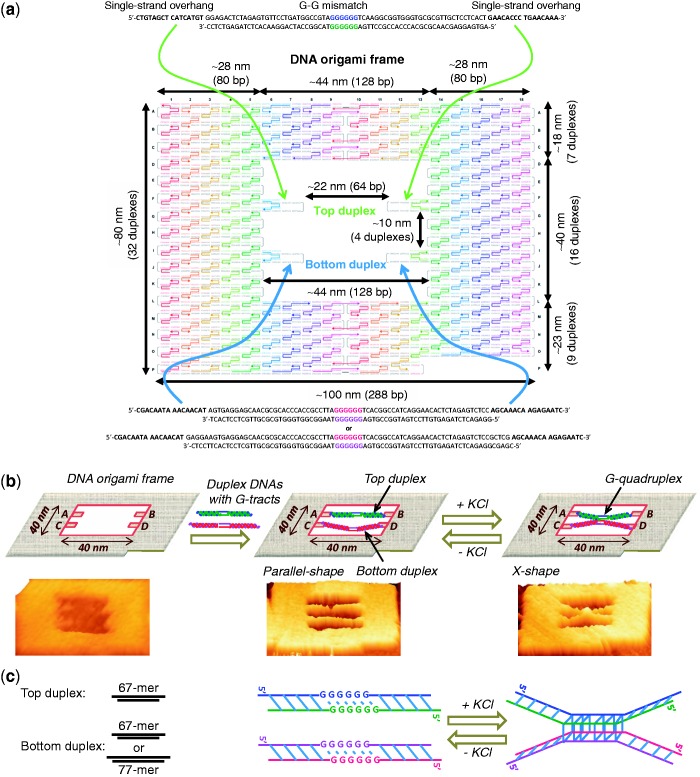
(**a**) The design of the DNA origami frame and the duplexes. (**b**) Graphical explanation of the DNA origami frame, insertion of the G–G mismatch repeats containing duplexes inside the frame and salt-mediated conformational changes of the incorporated strands. AFM image in each case is also given below the scheme. (**c**) Left: The length of the top duplex was 67-mer, while the bottom duplex used was either 67-mer (short duplex) or 77-mer (long duplex). Right: Schematic explanation of the DNA synapsis via the formation of G-quadruplex induced by KCl and the reverse conformation switching by the removal of the salt.

We have recently developed a defined DNA nanostructure, denoted as a ‘DNA origami frame’ (Figure 1a and b), to visualize the enzymatic reactions on double-stranded DNA (46,47). We used the same origami frame in the present study to observe the conformational switching and DNA synapsis with control over the stoichiometry and strand polarity. Briefly, this frame contains an inner vacant space of about 40 × 40 nm in which two sets of connection sites (A–B and C–D) were introduced to hybridize the duplex DNAs of interest. The length of each connection site is 32-mer (∼11 nm). The space between two connection sites (for example, A and B) is designed to be 64-mer double-stranded DNA, which corresponds to a length of ∼22 nm. To identify the orientation of the origami frame, a lacking corner at the right bottom of the frame was introduced (see Figure 1a and b).

For the G-repeat sequences, we have designed two different unique DNAs that are duplexes containing the G–G mismatch repeats in the middle. In each duplex, one of the strands is designed to have 16 bases long single-stranded overhangs at both termini that can assist the catenation of the duplex at the connecting sites present in the origami frame. To investigate the effect of the number of G-repeats on the formation of G-quadruplex, we adopted three, four or six G-repeats in each strand while keeping the number of base pairs of the duplexes constant (Supplementary Table S1). Because we fixed the duplex DNAs of interest inside the DNA origami, they may not diffuse easily to form the intermolecular G-quadruplex structure. Thus, to bring the duplexes closer, we imposed the structural flexibility to the incorporated strands by increasing the number of base pairs. Hence, the length of the top duplex was kept constant at 67-mer, the bottom duplex was varied to either 67-mer (‘short duplex’) or 77-mer (‘long duplex’) and the length between the two connecting strands in the origami was 64-mer (Figure 1c). The sequences used in this study are listed in Supplementary Table S1.

### Preparation of the origami assembly with synapsable duplexes

The origami frame was fabricated by folding the M13mp18 viral genome using 226 staple strands (32). The top and bottom duplexes were also prepared separately under similar conditions. A 10-fold excess of each duplex was mixed with the origami frame. The second annealing yielded the final duplex assembly inside the origami frame. The HS-AFM analyses were performed after removing the excess amount of unbound duplexes and staple strands in solution (for further details, see experimental section).

### Static observation of the G-quadruplex formation

**Figure 2.**
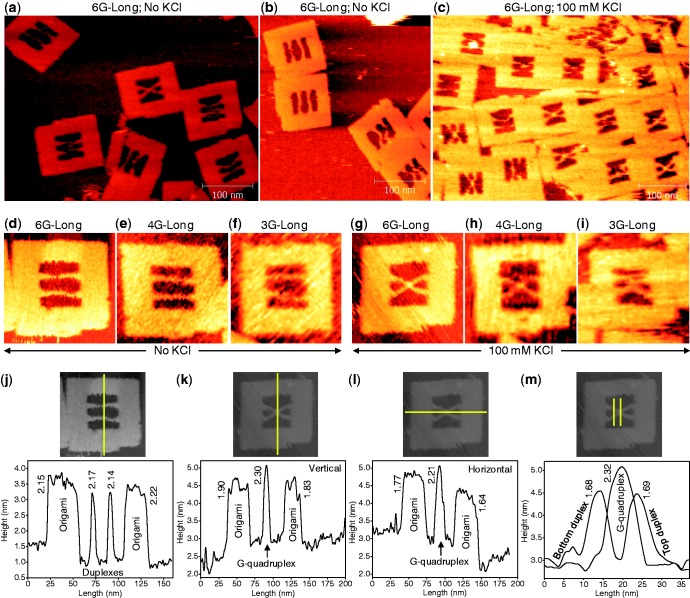
The Zoom-out AFM images of the DNA origami frame with incorporated duplexes recorded in the absence (**a, b**) and presence (**c**) of KCl for the G-repeat number of six. The parallel-shape of the incorporated strands can be clearly seen in the absence of KCl, indicating that no G-quadruplex is formed in this case. The X-shape in the presence of KCl evidences the formation of the quadruplex structure. The representative zoom-in images recorded in the absence of KCl for the sequences that contained six (**d**), four (**e**) and three (**f**) G-repeats. The same sequences in the presence of KCl are given, respectively, in (**g–i**). (**j**) The height profile estimated from the image given above the graph indicates that the origami frame and the incorporated duplexes are nearly same in height. (**k–l**) Height profiles estimated (vertical: k, and horizontal: l) indicate that the X-shape is slightly taller than the origami frame. This could be due to the formation of the four-stranded G-quadruplex which is taller than the duplexes in origami. (**m**) The height profiles of the duplexes and G-quadruplex regions. Yellow lines in the images indicate the locations where the height analyses were performed. The numbers in the graphs represent the estimated heights in nm of the peaks from the respective base line. All the images given in this figure were recorded for 67-mer top and 77-mer bottom duplexes. Image size: 125 × 125 nm (d–m). [KCl] = 0 or 100 mM; [MgCl_2_] = 10 mM; [Tris-HCl] = 20 mM, pH 7.6.

We first characterized the topology of the long duplex system with six G-repeats. Under K^+^-free condition, the incorporated strands laid parallel to each other, and both the duplexes could be seen clearly in the AFM images (Figure 2a and b). Similar results were also obtained for all other sequences with different G-repeat numbers (Figure 2d–f, and Supplementary Figure S1). Next, we performed the analyses under K^+^ environment. Interestingly, the topology of the incorporated duplexes changed and adopted an X-shape by stably binding the double helices in the middle of the duplex, as shown in Figure 2c. This topology change in the presence of K^+^ may be due to the formation of the G-quadruplex structure from the G–G mismatches present in both duplexes. Such a topology change and the formation of the X-shape were observed for all three G-repeat sequences tested, as seen in the AFM images in Figure 2g–i (also Supplementary Figure S1). The duplex regions of the incorporated strands can be seen clearly in the AFM images even after the formation of the X-shape.

### Height and width profiles

In the absence of K^+^, the estimated height difference between the origami frame and the incorporated duplexes was nearly zero for the long duplex with six G-repeats (Figure 2j). This is because both the origami frame and the duplexes adopted the B-form conformation and hence displayed the same height profile. However, in the presence of K^+^, the strands formed an X-shape, and the height analysis indicated that the X-shape was ∼0.6 nm taller than the origami frame (Figure 2k and l). Moreover, the height profiles estimated for the duplex and in the middle of the X-region of the incorporated strands in the same AFM image also indicated a taller profile of ∼0.6 nm for the X-region, as shown in Figure 2m. It has been shown that different types of G-quadruplex structures [quadruplexes formed within a longer duplex DNA with loops, blobs and spurs (58), and closely spaced G-quartets as in the case of G-wires (59) or loosely spaced G-quartets as in the beads-on-a-string structure (59)] display different heights; however, all are slightly taller (≥0.5 nm) than the duplex DNA. Thus, our findings indicated that the X-shape was produced by the formation of the four-stranded G-quadruplex, which is slightly taller than the two-stranded origami frame or incorporated duplexes.

We also calculated the difference in width between the duplex strands and X-region, and the latter was ∼2.1 nm wider than the duplexes (Figure 2m). This indicated the association of four strands in middle of the X-region, possibly because of the formation of G-quadruplex structure. Note, the G-quadruplex region is tiny and our estimation may have a positional error, and it is uncertain whether we have exactly estimated the width on the G-quadruplex or on the merging of the duplex strands. However, our estimation indicates that the duplexes are in close proximity and it might be due to the formation of the G-quadruplex. The difference in height between the duplex regions and origami frame was found to be nearly zero even after the quadruplex formation, indicating that the quadruplex formation allowed the DNA synapsis without disturbing the duplex regions of the participating sequences. The most reliable dimension in the AFM technique is the height value. Here, the estimated height values agree well with the original design typical for B-form DNA and G-quadruplex structure. The width values were slightly overestimated because of the common problem of tip-sample convolution in the AFM technique. However, we have considered the difference in width and the errors might have subtracted already and obtained the meaningful values.

### Statistical analysis

To obtain statistically meaningful values, we calculated the yield of the parallel- and X-shapes under K^+^ and K^+^-free environments for all possible combinations of strands with different G-repeat numbers and length (Table 1). In case of long duplex (67-mer top and 77-mer bottom duplexes) with six G-repeats, most of the duplexes (77%) adopted the parallel-shape, whereas only a minor but significant amount of duplexes (23%) were found to form the X-shape when no K^+^ ion was added. This indicated that, in the absence of K^+^, most of the incorporated strands did not form the G-quadruplex. An abrupt change in the ratio between the parallel- and X-shapes was observed when K^+^ was added, where 76% of the X-shapes and 24% of the parallel-shapes were observed, indicating the formation of the G-quadruplex. Similar results were obtained for short duplex (both top and bottom duplexes were 67-mer) in which 14% and 41% of X-shapes were found to exist in the absence and presence of K^+^ ions, respectively. The analyses with G-repeats of four and three yielded similar trends, and in all the cases, K^+^ induced the formation of the G-quadruplex structure.

**Table 1.** The yield (%) of the X-shapes calculated for all possible combinations of the strands

Sequences	No KCl		100 mM KCl	
	Long	Short	Long	Short
6G	23 (374)	14 (333)	76 (276)	41 (203)
4G	21 (333)	8 (381)	45 (248)	26 (208)
3G	13 (132)	6 (371)	31 (147)	15 (152)

The terms long and short represent 77 - and 67-mer bottom duplexes, respectively. The length of the top duplex was 67-mer in all cases. 6 G, 4 G and 3 G represent the number of contiguous guanines. The % yield of the parallel-shape = 100 − (yield of X-shape). The numbers in the parenthesis indicate the number of origami tiles counted in each case. [KCl] = 0 or 100 mM; [MgCl_2_] = 10 mM; [Tris–HCl] = 20 mM, pH 7.6.

**Figure 3.**
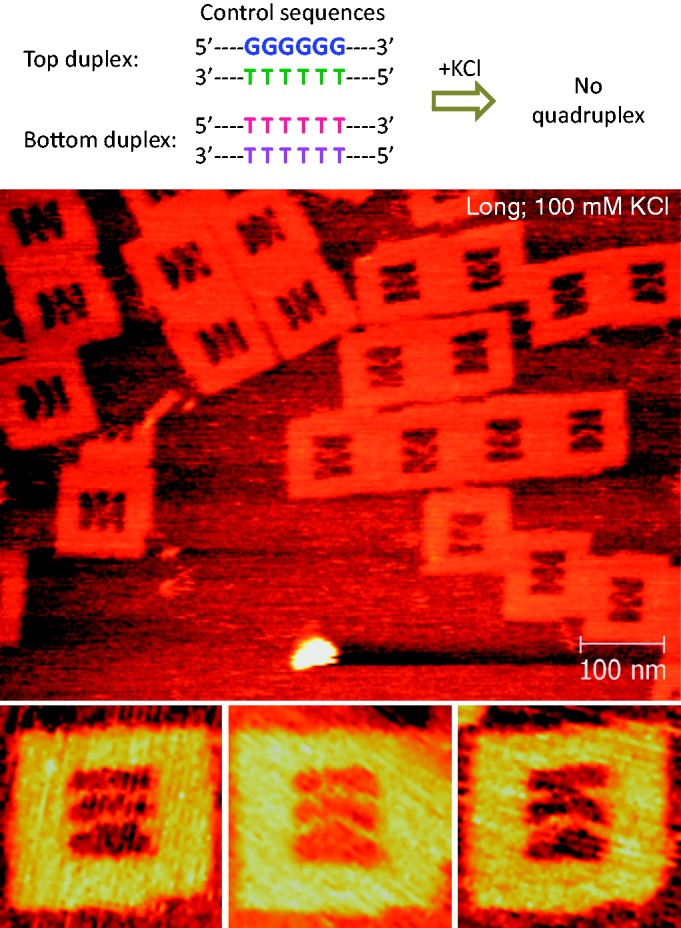
Control experiment with six G–T (top duplex) and T–T (bottom duplex) mismatch-containing duplexes in the presence of 100 mM KCl. Long duplex system was used. The mismatch regions are shown above the images, and other regions in the sequence are same as in Figure 1a. The absence of X-shape indicates the absence of the quadruplex structure. Zoom-in image size: 125 × 125 nm. [KCl] = 100 mM (both in origami solution and observation buffer); [MgCl_2_] = 10 mM; [Tris–HCl] = 20 mM, pH 7.6.

In addition to the yield values, we derived the following information from our analysis: (i) the longer the G-repeats, the higher the G-quadruplex formation (i.e. 6 G > 4 G > 3 G); (ii) the longer the length of the incorporated strands, the higher the G-quadruplex formation (i.e. 77-mer > 67-mer bottom duplex), indicating the need for the structural flexibility of the incorporated strands for the formation of the G-quadruplex; (iii) because we fixed both duplexes inside the origami frame and the strands within a duplex are oriented antiparallel, the strand polarity of the formed G-quadruplex is expected to be antiparallel (Note, the imposed structural flexibility to the incorporated strands are just to bring the duplexes closer, so that they can form the quadruplex structure at the middle where the G–G mismatches are present. However, this structural flexibility is not sufficient for the strands to form other type of structures such as parallel G-quadruplex. There may be a possibility for the formation of mixed G-quadruplex conformation between the antiparallel duplexes in which two strands orient in one direction and other two in opposite direction.); (iv) the G-quadruplex was formed at the center of the incorporated strands, while leaving the duplex regions unaltered; (v) we could successfully monitor the conformational switching of the G-rich strands with as few as three contiguous G-repeats with concentrations in the nanomolar range of the strands in solution and roughly picomolar on the mica surface; (vi) in all cases, a minor but significant amount of X-shapes were formed in the K^+^-free condition, possibly 10 mM Mg^2+^ present in the buffer may have induced a small amount of quadruplex structure. Note that previous studies using electrospray ionization mass spectrometry proved that the G-quadruplex can be formed in the presence of alkaline earth metal ions such as Mg^2+^, Ca^2+^, Sr^2+^ and Ba^2+^ (48). The stability order obtained was Sr^2+ ^> Ba^2+ ^> Ca^2+^ >> Mg^2+^ (48). Thus, we expect that the X-shape in the K^+^-free buffer could be the G-quadruplex induced by Mg^2+^ ions. This is also evidenced by the slight decrease in the X-shape (18% out of 331 frames counted and 17% out of 220 frames counted for the long duplexes with six and four contiguous guanines, respectively) at a lower Mg^2+^ concentration of 5 mM. Further, in some cases, the duplexes may be anchored closely and firmly on the mica surface and may look like an X-shape rather than the true formation of the G-quadruplex. Moreover, control experiment with six contiguous G–T and T–T mismatch-containing duplexes failed to produce the X-shape in the presence of 100 mM of K^+^ (2% X-shapes were found out of 273 frames counted, Figure 3), indicating that the minor amount of X-shape observed above in the absence of K^+^ is due to the Mg^2+^-induced quadruplex structure. This control experiment further evidences that the X-shapes obtained for the G–G mismatch-containing sequences is because of the formation of the G-quadruplex structure.

### Real-time observation of the salt-induced formation of a G-quadruplex

One of the major goals for scientists in various disciplines is to analyze the biological reactions and functions in real-time (60–62), which could offer new insights. Real-time analysis is not possible with most available techniques, and HS-AFM is one of the few techniques available (49). Although the real-time analysis described below does not provide all the possible information, it is in fact a good initiation.

**Figure 4.**
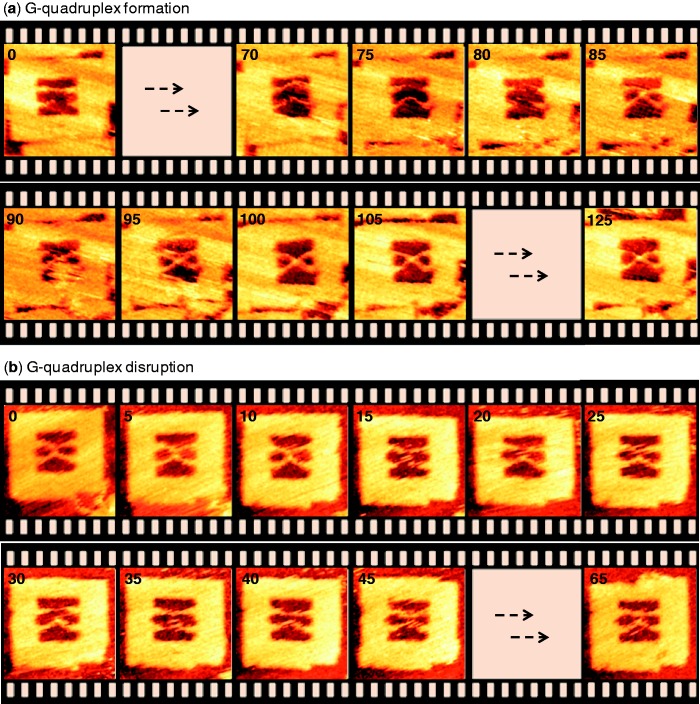
The snapshots of the real-time HS-AFM imaging of the conformational changes. (**a**) Salt-induced formation of a G-quadruplex. The origami was prepared and immobilized on mica surface in a KCl-free buffer, while the imaging was carried out in a buffer that contained 100 mM KCl. (**b**) The deformation of a G-quadruplex structure under KCl-free environment. The origami was prepared and immobilized on mica surface in a buffer containing 100 mM KCl, whereas the observation buffer contained no KCl. The long duplex system (67-mer top and 77-mer bottom duplexes) with six G-repeats was used in these studies. The numbers at the top left corner represent the imaging time in second. Image size: 125 × 125 nm; scan speed: 0.2 frame/s. [MgCl_2_] = 10 mM; [Tris–HCl] = 20 mM, pH 7.6. For real-time movies, see Supplementary section.

To visualize directly the formation of a single G-quadruplex in real-time, we selected the long duplex with six contiguous guanines as a representative example. The duplexes-incorporated origami frame was prepared, purified and adsorbed onto a freshly cleaved mica plate in the absence of any added K^+^. The sample was scanned in an observation buffer that contained 100 mM K^+^, and the image acquisition frequency was 0.2 frame/s. The results of the HS-AFM imaging are summarized in Figure 4a, and the real-time movie is given in Supplementary Movie S1. During the imaging, the incorporated duplexes laid parallel to each other and maintained the parallel-shape until 80 s, after that the strands suddenly (i.e. in ≤5 s) formed an X-shape. Once the X-shape was formed, it remained unchanged and was not dissociated throughout the scanning (125 s). This indicated clearly that the X-shape resulted by the conformational change from the G–G mismatch strands to a G-quadruplex and not due to a simple overlap of the duplexes.

The duplex regions seemed not to be disturbed throughout the imaging, indicating that the DNA synapsis via G-quadruplex formation takes place without altering the duplex regions of the G-rich strands. In general, the quadruplex structure may be formed within the time scale of few milliseconds to 0.2 s, depending on various factors such as sequence, number of G-repeats and concentration of salt (63). Here, we could discern that the changes occurred within 5 s because the scan rate was 0.2 frame/s. However, the rate of the quadruplex formation within the origami scaffold on a mica surface may be slower than in bulk solution where the G-strands may have higher diffusion coefficient in the latter case (17). On the other hand, confining the duplexes within the nanospace increases the local strand concentration, which may increase the association rate. The conformational change from the parallel- to the X-shape was often observed, and we could reproduce such a conformational change in real-time (see Supplementary Figure S2).

### Real-time analysis of the disruption of a G-quadruplex

Similar to the formation event, we monitored the deformation of a single G-quadruplex in the absence of K^+^ ions for the same sequences. The origami frame with duplex strands of interest was prepared, purified and immobilized on a mica surface in a buffer containing 100 mM K^+^. The scanning was performed by immersing the mica surface in K^+^-free observation buffer. This K^+^-free buffer is expected to remove the K^+^ that are prebound to the G-quadruplex and consequently destabilize it. As expected, the X-shape was observed at 0 s and remained unaltered for 10 s (Figure 4b, see Supplementary Movie S2 for the real-time movie). Then, the deformation of the X-shape was observed and resulted in the parallel-state of the G–G mismatch duplexes. Once the X-shape dissociated into the parallel-shape, the reverse conformational switching of the parallel- to X-shape was not observed thereafter (up to 65 s), indicating the deformation of the G-quadruplex by the release of K^+^ ions. As in the case of G-quadruplex formation, the duplex regions were unaffected by the deformation of the DNA synapsis.

Real-time analysis has several advantages over static observation. The dynamics of a single G-quadruplex formation or disruption can be monitored only by real-time analysis. Using the static analysis, it is difficult to estimate the time required for the quadruplex formation or deformation, whereas at least a rough estimation is possible in the real-time analysis.

In conclusion, we report here the direct observation and single-molecule analysis of the KCl-induced formation of a tetramolecular G-quadruplex within a DNA nanoscaffold. The conformational switching of the G–G mismatched sequences within the duplex DNAs to a G-quadruplex formation was observed by using HS-AFM by monitoring the topology change of the strands. In a K^+^-free buffer, most of the incorporated duplexes had no interaction and laid parallel to each other. Addition of K^+^ induced the formation of a G-quadruplex structure by stably binding the double helices to one another in the middle of the duplexes. Such a quadruplex formation allowed the synapsis of the duplex DNAs, and the duplex regions of the participating sequences were found to be unaltered throughout our investigations. The effects of the duplex length and the number of G-repeats on the formation of the G-quadruplex structure were also investigated. We could monitor the formation of the G-quadruplex structure with a G-repeat number of as low as three and with a concentration of the strands in nanomolar range in solution and picomolar range on the mica surface. The G-quadruplex formation in a buffer solution containing K^+^ and its deformation in K^+^-free buffer were analyzed in real-time. The orientation of the G-quadruplex is often difficult to control using traditional biochemical methods, particularly in the case of the intermolecular G-quadruplex structure. Because the G–G mismatched sequences were fixed inside the origami frame and the strands within a duplex were oriented antiparallel, the strand polarity of the formed G-quadruplex could be controlled. Moreover, the strand stoichiometry could also be controlled and is exclusively four in the present case. We have recently reported the ability of the DNA origami structures for the analysis of the DNA conformational changes such as (3 + 1) type G-quadruplex (17) and B–Z conformational transition (38). Here, we add one more example of the DNA conformational changes by investigating the formation and disruption of the intermolecular four-stranded G-quadruplex within a DNA origami nanoscaffold. We expect that our method can be useful for understanding the fundamentals of other conformations of nucleic acids, drug screening and protein-induced conformational switching.

## SUPPLEMENTARY DATA

Supplementary Data are available at NAR Online.

## FUNDING

CREST, Japan Science and Technology Corporation (JST); WPI program, WPI-iCeMS, Kyoto University; Grant-in-Aid for Scientific Research, the Ministry of Education, Culture, Sports, Science and Technology (MEXT), Japan; Asahi Glass Foundation (to M.E.); Japan Society for the Promotion of Science (JSPS) Postdoctoral fellowship (to A.R.); Aquitaine Regional Council and the ANR program F-DNA, Quarpdiem and Oligoswitch (to P.L.T.T. and J.-L.M.). Funding for open access charge: CREST, JST.

*Conflict of interest statement.* None declared.

## Supplementary Material

Supplementary Data
