# Supplementary material for: Controlling the stoichiometry and strand polarity of a tetramolecular G-quadruplex structure by using a DNA origami frame
Source: Nucleic Acids Res. 2013 Jul 17;41(18):8738–47. doi: 10.1093/nar/gkt592 (PMC3794576; doi:10.1093/nar/gkt592)
Supplement: Supplementary Data [file supp_41_18_8738__index.html]

Controlling the stoichiometry and strand polarity of a tetramolecular G-quadruplex structure by using a DNA origami frame — Controlling the stoichiometry and strand polarity of a tetramolecular G-quadruplex structure by using a DNA origami frame — Supplementary Data 

# Controlling the stoichiometry and strand polarity of a tetramolecular G-quadruplex structure by using a DNA origami frame

## 

files

**Files in this Data Supplement:**

- Supplementary Data - doc file
