# Supplementary material for: Controlling the stoichiometry and strand polarity of a tetramolecular G-quadruplex structure by using a DNA origami frame
Source: Nucleic Acids Res. 2013 Jul 17;41(18):8738–47. doi: 10.1093/nar/gkt592 (PMC3794576; doi:10.1093/nar/gkt592)
Supplement: Supplementary Data [file supp_gkt592_nar-01339-f-2013-File002.doc]

***Supplementary Information***

**Controlling the stoichiometry and strand polarity of a tetramolecular G-quadruplex structure by using a DNA origami frame**

Arivazhagan Rajendran,^1^ Masayuki Endo,^2,3,*^ Kumi Hidaka,^1^ Phong Lan Thao Tran,^4^ Jean-Louis Mergny,^4^ and Hiroshi Sugiyama^1,2,3,*^

^1^Department of Chemistry, Graduate School of Science, Kyoto University, Kitashirakawa-oiwakecho, Sakyo-ku, Kyoto 606-8502, Japan.

^2^Institute for Integrated Cell-Material Sciences (WPI-iCeMS), Kyoto University, Yoshida-ushinomiyacho, Sakyo-ku, Kyoto 606-8501, Japan.

^3^CREST, Japan Science and Technology Corporation (JST), Sanbancho, Chiyoda-ku, Tokyo 102-0075, Japan.

^4^Univ. Bordeaux, INSERM, U869, ARNA Laboratory, 2 rue Robert Escarpit, Pessac, F-33607, France.

*To whom correspondence should be addressed. Tel.: (+)81-75-753-4002; Fax: (+)81-75-753-3670.

E-mail: endo@kuchem.kyoto-u.ac.jp (M.E.); hs@kuchem.kyoto-u.ac.jp (H.S.)

Present address: Phong Lan Thao Tran, Department of Molecular Biology, Princeton University, Princeton, NJ 08544, United States.

**Note on the movies:** The image acquisition speed was 0.2 frame/s. However, the frame rate in the movies is 5 times speed enhanced (1 frame/s). Image acquisition time is given at the top left corner in each frame. Image size: 125 × 125 nm.

Table S1. The sequences used in this study

Figure S1. Zoom-out images of the origami assembly with long duplexes containing four (a-b) and three (c-d) contiguous guanines. White arrows indicate the X-shape of the incorporated strands. [Tris-HCl] = 20 mM, pH 7.6; [MgCl_2_] = 10 mM; [KCl] = 0 or 100 mM.

Figure S2. Additional HS-AFM images of the real-time analysis of the salt-induced formation of a G-quadruplex. The long duplex system (67-mer top and 77-mer bottom duplexes) with six G-repeats was used in this study. The origami was prepared and immobilized on mica surface in a KCl-free buffer, while the imaging was carried out in a buffer that contained 100 mM KCl. The numbers at the top left corner represent the imaging time in second. Image size: 150 × 150 nm; scan speed: 0.2 frame/s. [Tris-HCl] = 20 mM, pH 7.6; [MgCl_2_] = 10 mM; [EDTA] = 0 (observation buffer) or 1 mM (origami solution).
